# Socio-spatial inequalities and the distribution of cash benefits to asthmatic children in Norway

**DOI:** 10.1186/1476-072X-8-22

**Published:** 2009-04-23

**Authors:** Jon Erik Finnvold

**Affiliations:** 1 Statistics Norway, Kongens gate 6, PO Box 8131 Dep, NO-0033 Oslo, Norway

## Abstract

**Background:**

Previous research has observed large inequalities in the distribution of welfare benefits. The Norwegian welfare state includes several schemes that give households with children the opportunity to apply for public income support to compensate for expenses related to chronic disease. The aims of this study were to examine the geographic distribution of children receiving compensatory cash benefits because of a chronic asthma condition and to determine whether social or geographic factors account for area variations in uptake independent of the associations with need.

**Results:**

Considerable variation between counties was evident, with rates of benefit uptake ranging from 10.5 recipients per 1,000 children younger than nine years in the highest-ranking county, to 1.5 per 1,000 in the lowest. It is argued that the observed area-level inequalities reflect more than variations in morbidity. In particular, the chance of receiving benefits reflects variations in the ability of street-level bureaucrats to inform families about their rights. Spatial variations also reflect, in part, the fact that families with different socio-economic standing inhabit different locations and that the threshold for receiving benefits is systematically lower for families with an academic background.

**Conclusion:**

To be able to refine the implementation of a welfare policy, a better understanding of the processes that generate the outcomes of the various welfare schemes and services is required. This can be achieved by adopting an approach to the study of outcomes of welfare policy that integrates both the social and geographic perspective, and that focuses on specific diagnoses or distributional procedures.

## Background

The Norwegian welfare state includes several schemes that give households with children the opportunity to apply for public income support to compensate for expenses related to chronic disease. These benefits cover normal daily expenses (basic benefit) and expenses for the care and nursing of the child at home (attendance benefit). Basic and attendance benefits (BA-benefits) are based solely on the presence and severity of illness. Benefits are in no way dependent on the financial means or other social qualifications of the family. The aims of this study were to examine the geographic distribution of children receiving BA-benefits and to determine whether social or geographic factors account for area variations in uptake independent of the association with need.

To become a recipient, an applicant family must meet several necessary conditions. First, an application must be forwarded to the local insurance administration. This application must include medical documentation provided by a doctor asserting that the child has a chronic condition that remains even after an adequate treatment programme is established. The final decision is taken by the local executive officers who decide on how to apply the rules of the national political authorities in each individual case. If the distributional process accords with its political intention, observed area-level variations are assumed to reflect variations in morbidity only. However, no formal institutional arrangement is oriented toward informing families with a child with a chronic illness about their rights to benefits, and there is no mechanism that secures children with a qualified condition actual access to the benefits. Less is known about how families become aware of the possibility of applying for benefit, and what role physicians, nurses and other welfare professionals play in the process. Only those who have made a successful claim are included in the official register of recipients.

The central health authorities in Norway annually publish information about the regional distribution of children receiving BA-benefits [1]. It is believed that regional differences in the uptake of benefit in part can resemble differences in the prevalence of disease, and that documentation of area-level variation in uptake can be used to identify areas with need for intervention. In a report published by The National Insurance, substantial and unexplained regional differences in uptake of BA-benefit are observed [2]. The report concluded that differences to a significant extent result from variations in the tendency to apply for benefit.

Research based on register analyses from the UK have also suggested that area-level variations in uptake of health related benefits might be related to the ability to make successful claims [3]. The international literature on cash benefit has to a large extent focused on the role of physicians and other professional groups in the application process [4,5]. However, the experiences of the recipients have to a lesser extent been investigated.

The present paper focus on the experiences of one group of recipients: parents with asthmatic children. Children with asthma are the largest of the diagnostic groups that are recipients of BA-benefits. A previous investigation has documented considerable area differences in uptake for this particular group [6]. Research also suggests that a gradient exists with regard to the social distribution of the burden of asthma: morbidity rises with decreasing parental education [7-10]. Social inequalities in heath also have implications for area variations in the distribution of BA-benefit. Another argument in favour of the decision to focus on one diagnosis only is based on methodological considerations. When conducting surveys that include only one diagnosis, it is possible to differentiate the children according to the severity of the disease in a manner not possible if the sample include different diagnoses.

This empirical investigation identifies as its point of departure previously documented regional variations in the uptake of benefits for children with asthma [6]. The overall aim of the paper is to explore possible mechanisms that contributes to the production of regional variations in uptake. Using a survey of parents of all children receiving benefits, this study investigated the parents' experiences of the process of applying for benefits and important aspects of the children's health condition.

The following questions were asked.

• Do children in regions with a high level of uptake have a less serious asthma condition, suggesting that the threshold for receiving benefits is lower?

• Do parents in regions with a high level of uptake have a more positive overall evaluation of the application process?

• Is there a spatial element in the organization of health care that influences the way parents experience the application process?

• Are parents with an academic background less likely to report delays in the process of applying for benefits? If so, to what extent can differences in the settlement pattern of parents with an academic background account for the observed regional differences?

• Do parents with an academic background have children with a less severe asthma condition, suggesting that the threshold for receiving benefit is lower?

The findings are discussed in relation to the capacity of universal welfare programmes to distribute needs-tested financial resources in a way that achieves equity. By focusing on area variation, the analysis provides a more general insight into the processes that lead to access to health-related cash benefits. The empirical analysis is introduced by a conceptual investigation of the mechanisms that cause families with a child with chronic illness to apply for benefits.

### Area inequalities and social inequalities

The chance of receiving benefits may vary across space for several reasons. Part of this variation is a pure compositional effect: people with different socio-economic standing may have different levels of morbidity or different levels of uptake of the benefit. Socio-economically similar families also tend to cluster in space [11,12]. Spatial variations may thus emerge simply because different groups inhabit different locations.

Several implications for area variation follow from the fact that people with different socio-economic standing are segregated regionally. First, a case can be made for a situation in which a high concentration of highly educated or affluent families may lead to a lower uptake of benefits. This expectation is based on evidence from Norway and other countries suggesting that asthma is more frequent within more depressed regions and within the less affluent and educated segment of the population [7-10].

It can also be argued that, in general, highly educated social groups are more likely to succeed in their dealings with welfare professionals. Within the research on the welfare state, the term "Matthew effect" has been applied to identify situations in which those already in a position of advantage are better able to extend that advantage or to enjoy disproportionate rewards compared with the disadvantaged [13,14]. One possible explanation for the observed "Matthew effect" is that providers treat social groups differently. For example, a previous overview of the literature on provider behaviour in medical encounters concluded that physicians give more information to patients from higher social classes [15].

The direction of causality may also be the opposite. There is evidence that groups with higher socio-economic standing have a better ability to influence the results of exchange relations with bureaucrats or practitioners. This is partly because clients from the more educated segment of the population are better informed about the workings of the service system and how benefits can best be obtained. In general, groups with higher socio-economic standing are characterized by more developed social networks [16]. Available evidence also indicates that these groups are more inclined to seek membership in organizations or to participate in organizational activities [17]. It follows that people with an academic background will tend to have more personal contacts with welfare professionals. It is also more likely that families with higher socio-economic standing will have membership in patient organizations. All of these factors may increase their knowledge of social rights attached to chronic illness relative to other social groups.

Evidence also suggests that groups with high income and education are more likely to be attracted to more specialized or privately funded medical services [18]. This can have implications for their chances of receiving benefits (see below). However, income is less likely to be a barrier, as health care in Norway are publicly funded, including paediatricians who practice on a private basis.

### Informal lay-referral systems

A distinction can be made between characteristics of a locality or area that have an impact on peoples' awareness of social rights associated with chronic disease (informal lay referral systems) and factors associated with the fact that people in different places face different formal institutional environments (characteristics of the street-level bureaucracy). The former presupposes an element of social interaction between a family and their surrounding social networks. The latter is determined by larger structural forces in that local or regional jurisdictions offer different kinds of health care facilities and services. Private specialist care, for instance, is heavily concentrated in urbanized areas. Such variations are bound to affect people's access to cash benefit schemes.

Decisions to visit health care facilities or to explore possibilities for applying for compensating benefits related to illness are often made privately by the individual. However, a great deal of evidence suggests that wider social processes influence the way people deal with the health and social care system. Patients may organize themselves informally and form local self-help groups [19]. Formal patient organizations also exist at a national level with local branches. One of the tasks of these organizations is to spread information about available benefits and services offered by the welfare state. These organizations also offer social activities through which people can meet and exchange experiences and information. Membership and participation in these organizations thus influence peoples' knowledge about the rights and entitlements attached to chronic disease. Both the size and character of individuals' social network may affect their awareness of the social rights attached to chronic disease. Most obviously, the presence of a physician within this network may have consequences for their access to health-related goods and services. Physicians in general are not necessarily well informed about welfare benefits, but knowing a physician may nevertheless increase the chances of becoming a recipient.

### Characteristics of street-level bureaucracy

People in different places face different formal institutional environments. Street-level bureaucrats are public service workers who interact directly with citizens, and have substantial discretion in the execution of their work [[14], p. 3–4]. Street-level bureaucrats within the local insurance administration play important roles by helping people complete forms, informing the public about their rights and giving information to the health care institutions within their municipality about their role in the application process. Local traditions or specific cultures within insurance organizations may cause variations in the tendency to award benefits. For example, local insurance officers may have different interpretations of the rules defining eligibility [20].

Physicians and other professional groups within the health services also play key roles in the application process for benefits. Physicians may differ in their decisions on whether or not to sickness certify patients in consultations [4,5]. Most families with a child with chronic disease have a regular site of care and a regular doctor. The most common sites of care used by children with severe asthma include a specialist at a hospital or a private practicing specialist. Specific characteristics of such sites may influence the behaviour of both doctors and clients. Freidson [21] distinguished between "patient-dependent practice" (solo practitioners) and "colleague-dependent practice" (more complex organizational forms). Everyday medical settings are distributed along a continuum between these two forms. According to Freidson, the patient-dependent practice is inherently unstable. To maintain a sustainable practice, the doctor must recruit a reasonable pool of patients, and to keep them, he must give them what they want ... or someone else will [[21], p.92]. It may be argued that the incentive to inform patients about their social rights differs between the private practice context and the hospital ward, or that private practicing paediatricians have a different orientation toward benefits. The doctors in hospital settings are not responsible for recruiting their own patients because patients are either referred to the hospital by lower levels in the medical hierarchy or admitted during acute situations. Specialists in a hospital setting are dependent on the career possibilities offered to them within the hospital system.

In addition, the distinction between patient-dependent and colleague-dependent practice in this case coincides with the private and public modes of service provision. There is reason to believe that the symmetry and mutual dependence between a family and their doctor differs between the private and public context [22]. The expectation is that uptake is likely to increase when the patient-centred private institutional context dominates. In Norway, the availability of private practice paediatricians is characterized by a highly uneven geographic distribution [23]. Norwegian health care personnel are mainly salaried employees. One main exception is privately practicing paediatricians, who are paid by fee-for-service from The National Insurance, and lump sum grants from the regional health authorities [24]. The fee-for-service element in the financing of privately practicing paediatricians can imply a different mutual dependence between the family and their doctor compared to the situation in hospitals.

To allow the physician to concentrate only on somatic problems, some hospitals employ nurses or social workers to inform patients about their social rights. More applications for benefits are likely to be forwarded to the local insurance office in local hospital organizations whose functions are oriented toward informing patients and their families about social rights associated with chronic disease.

## Methods

The population of investigation was drawn from a national register containing information about recipients of the basic benefit and attendance benefit [25]. Such benefits are granted by the Norwegian National Insurance Administration. To be entitled to support, the child must have moderate to serious asthma, but milder conditions may qualify in special cases. Moderate asthma is defined as a condition where medication is required on a daily basis [[25], § 6-4]. The maximum amount of benefit per year that was given in 1997 was NOK 58,200, and the minimum was NOK 5,700. The mean was NOK 15,300, an amount that corresponded to about half of the combined parental mean monthly income after tax.

At the end of 1997, the register included information on 25,676 children under the age of 16 years. The population under investigation was defined by choosing all children under the age of nine years with a reported diagnosis of asthma at the end of 1997 (code 493 in the International Classification of Diseases, version 9). This resulted in a population of 2,819 children with asthma. Because 224 families had more than one child who received a benefit, the population was reduced to 2,564 families. In families with more than one recipient, information was obtained about the eldest child. The decision to limit the population to children under the age of nine years was based on the assumption that the parents are the main actors and sources of information about the experiences of the early phase of the disease.

### Survey

The processes involved in uptake of benefits were addressed directly in a survey of all recipients of BA-benefits. Based on focus group interviews and in-depth interviews with parents with relevant experiences, a questionnaire was developed and a postal survey was carried out during the winter and spring of 1999. The questionnaire was returned by 1,800 families, representing a response rate of 70%. For most of the items in the questionnaire, the non-response rate was less than 2%.

The decision to focus on one diagnosis allowed for a more detailed examination of variations in morbidity in the population of BA-recipients. Accordingly, the parents' assessments of the general condition of their child were built into the questionnaire, as were the frequency of occurrence of asthma and sleep disorders. An overview of the variables is given in table 1, including details on the wording of questions included in the questionnaire. Variations in the families' experiences of the application process and the role played by professionals was also addressed in the survey, including information about the source of information about benefits, and the families' subjective evaluation of delays in the application process. Information about the social network included membership and participation in patient organizations and aspects of family networks such as close friends or relatives with a professional background, including physicians.

**Table 1:** Overview of empirical material

Variables	Data source
Basic/attendance benefit status: Recipients of cash benefit pr. 1000 household with children born 1989–1995, county averages. Norway consists of 19 county municipalities. The counties were grouped into four categories. 'Low' = 0–3.2, 'Low/medium' = 3.3–5.0, 'Medium/high' = 5.1–8.7 'High' = 8,8–10,5	National insurance [25]

Centrality: Least central municipalities.	Official population statistics [27]

Parents' education: Parents with the same educational level as physicians are represented within two groups in the analysis: one in which both parents have higher education, and the other in which only one parent has higher education. Higher education includes the first stage of tertiary education and postgraduate education, codes 7 and 8 in the Norwegian Standard Classification of Education. A category of parents with intermediate education includes codes 3, 4 and 5. This group includes parents with an educational level above compulsory education, including lower university level. The final group, lower education, includes compulsory education, codes 1 and 2.	Official register of education [26]

Regular source of care: 'Does the child use a regular doctor for the asthma problems?' Response categories: 'No, the child has no regular doctor', 'Yes, a general practitioner', 'Yes, a specialist/paediatrician outside hospital', 'Yes, a specialist/paediatrician at a hospital'.	Survey

Experiences of the process of application: 'How did you receive information about the possibility of applying for benefits?' Response categories: 'Through a doctor', 'Through a nurse or a social worker at a hospital', 'Through a social worker or other employees in the municipality, township or county office', 'Through The Norwegian Association for Asthma and Allergy or The Norwegian Association of Heart and Lung Patients', 'Through friends, family or other acquaintances', 'Other'	Survey
	
'Are you of the opinion that the application for benefit should have been sent before?' Response categories: 'No', 'Yes, we were not aware of our rights, 'Yes, other reasons for delay'.	

Social network and social participation: Are you a member of the Norwegian Association for Asthma and Allergy? Response categories: Yes/No Have you ever participated in arrangements or seminars on asthma, arranged by the Norwegian Association for Asthma and Allergy or other organizations? Response categories: Yes/No	Survey

Child's health condition: How do you assess his/her health-condition in general?' Response categories: 'Very good', 'Good', 'Fair', 'Bad', 'Very bad'.	Survey
	
'How many episodes of heavy breathing or wheezing in the chest has the child experienced during the previous 12 months?' Response categories: 'More than 12', '4 to 12', '1 to 3', 'None'.	
	
How many nights on average was the child's sleep interrupted because of cough during the previous 12 months? Response categories:'1 or more nights per week', 'Less than one night per week', 'Never woke up'.	

Information about the parents' education and residence was added from a national register [26,27]. This classification was based on the general expectation argued above that medical encounters between participants from the same social strata differ from encounters between participants with different educational backgrounds.

In the empirical investigation, one particular county (Akershus) is identified. This choice is based on findings from a previous investigation showing that Akershus had the highest rate of uptake [6]. Two additional arguments justify the inclusion of Akershus as a variable: the county was a preferred location for privately practicing paediatricians, and also had a higher proportion of affluent and highly educated families relative to other counties [28]. The remaining counties were grouped into four categories according to the level of uptake (table 1). Background variables also included regions with different levels of centrality.

Variables representing the child's health condition were used as outcomes. If a better health condition is observed for particular social groups or in particular regions, this may indicate that the threshold for receiving benefit is lower, and that a larger proportion of children suffering from asthma are granted benefit compared to other groups or regions. Experiences with delays in the process of application were used as an important outcome, details on the wording is presented in table 1.

The empirical significance of background variables was assessed by means of logistic regression analysis. First, the statistical effect of each variable was estimated separately. Second, a full model was estimated using multivariate analysis. The difference in significance and odds ratio of the regional variables in the first and second (multivariate analysis) column in the tables gives an impression of the degree to which the variables included in the second multivariate analysis can account for the statistical effect of the regional variables.

## Results

Considerable variation between counties was evident (Table 2); the rates of benefit uptake ranged from 10.5/1000 children (Akershus county) to 1.5/1000 children (Telemark county). The county of Akershus was the home 11 percent of all families with a child younger than nine years, and almost 23 percent of families with children who received cash benefits because of asthma lived in Akershus.

**Table 2:** Families with children born 1989–1995, N = 421 558 (Percent).

	Children	Children with benefit	Rates pr. 1000
All	547314 (99.8)	2801 (99,8)	5,1
			
Østfold	27533 (5.0)	132 (4.7)	4,8
Akershus	60722 (11.1)	637 (22.7)	10,5
Oslo	56521 (10.3)	271 (9.7)	4,8
Hedmark	20093 (3.6)	132 (4.7)	6,6
Oppland	19826 (3.6)	119 (4.2)	6,0
Buskerud	27128 (5.0)	124 (4.4)	4,6
Vestfold	24694 (4.5)	214 (7.6)	8,7
Telemark	18564 (3.4)	28 (1.0)	1,5
Aust-Agder	12261 (2.2)	93 (3.3)	7,6
Vest-Agder	20044 (3.7)	89 (3.2)	4,4
Rogaland	51970 (9.5)	110 (3.9)	2,1
Hordaland	56423 (10.3)	181 (6.5)	3,2
Sogn og Fjordane	13827 (2.5)	29 (1.0)	2,1
Møre og Romsdal	29724 (5.4)	60 (2.1)	2,0
Sør-Trøndelag	32692 (6.0)	162 (5.8)	5,0
Nord-Trøndelag	15866 (2.9)	120 (4.3)	7,6
Nordland	29673 (5.4)	96 (3.4)	3,2
Troms	19385 (3.5)	148 (5.3)	7,6
Finnmark	10272 (1.9)	56 (2.0)	5,5
Incomplete register observation	96	.	

Recipients of cash benefit with asthma, N = 2520 (Percent). Rates pr. 1000. County

Source: The national insurance and Statistics Norway

Table 3 and table 4 show descriptive statistics about the benefit recipients in Akershus and a comparison with the net sample including all families. The most noticeable difference is that 71% of families in Akershus reported a private specialist as their regular source of care compared with 31% in the net sample. Recipients in Akershus were also more likely to be informed by health personnel about the possibility of applying for benefits and less likely to report delays in the processing of the application. Staff at the local or regional insurance organizations seemed to play a minor role in informing families about the possibility of applying for benefits. Patient organizations were reported as the source of information by 6% of families and by 2% of the families living in Akershus. Participation and membership was less common in Akershus. Twenty-seven per cent of families in Akershus had a parent who had completed higher education, whereas the corresponding figure of the net sample was 22%. A slightly higher percentage of families in Akershus reported personal acquaintance with a doctor. Health conditions seemed to be better for recipients of BA-benefits in Akershus (table 4), but the differences were small.

**Table 3:** Experiences with the application for cash benefit

	Net sample (N = 1800)	Net sample minus Akershus (N = 1363)	Akershus (N = 437)
**"How did you receive information about the possibility of applying for benefits?"**			
1. Through a doctor	26	22	38
2. Through a nurse or a social worker at a hospital	10	9	14
3. Through a social worker or other employees in the municipality, township or county office	3	3	1
4. Through The Norwegian Association for Asthma and Allergy or The Norwegian Association of Heart and Lung Patients	6	7	2
5. Through friends, family or other acquaintances	29	32	21
6. Other^1^	5	5	4
7. Combinations of 1–6	22	22	22
Total	100	100	100
Item non-response	(12)	(9)	(3)
			
***Are you of the opinion that the application for benefit should have been sent before?***			
1. No	28	24	41
2. Yes, we were not aware of our rights	57	60	45
3. Yes, other reasons for delay	9	9	9
7. Combinations of 2 and 3	6	7	6
Total	100	100	100
Item non-response	(36)	(25)	(11)

Characteristics of net sample. Data are presented as percent. (N)

^1^Including health visitors and the media

**Table 4:** Physicians' everyday work setting, education, residence, social network and indicators of need-health condition

	Net sample (N = 1800)	Net sample minus Akershus (N = 1363)	Akershus (N = 437)
Regular source of care^1^			
Families using a private practice specialist as their regular doctor	31	18	71
Others	69	82	29
Total	100	100	100
Item non-response	(19)	(16)	(3)
			
Parents' educational background^2^			
Both parents higher education	6	5	9
One parent higher education	16	15	18
Intermediate education	60	60	60
Both parents lower education	18	13	13
Total	100	100	100
Missing data or incomplete register observation	(91)	(70)	(21)
			
Social network and social participation			
*Membership in the Norwegian Association for Asthma and Allergy*			
Yes	50	51	47
No	50	49	53
Total	100	100	100
Item non-response	(9)	(6)	(3)
*Participation in activities related to asthma arranged by patient organizations*			
Yes	36	39	29
No	64	61	71
Total	100	100	100
Item non-response	(17)	(11)	(6)
*Social network *Families with a close friend or relative who is a doctor			
Yes	5	4	7
No	95	96	93
Total	100	100	100
			
Indicators of relative need or health condition			
*The parents' reports of their children's health*			
Very good	11	10	14
Good	51	50	50
Fair	32	33	30
Bad/Very bad	7	7	7
Total	101	100	100
Item non-response	(23)	(19)	(4)
			
*Number of episodes of heavy breathing or wheezing in the chest during the previous 12 months*			
More than 12	25	27	18
4 to 12	51	51	49
1 to 3	21	19	28
None	3	3	5
Total	100	100	100
Item non-response	(14)	(11)	(3)
			
*How many nights on average was the child's sleep interrupted because of cough during the previous 12 months?*			
1 or more nights per week	35	37	30
Less than one night per week	56	55	59
Never woke up because of cough	9	8	11
Total	100	100	100
Item non-response	(13)	(11)	(2)

^1'^Others' include specialist at a hospital ward, general practitioner, or no regular source of care

^2^Higher education includes first stage of tertiary education and postgraduate education, code 7 and 8 in The Norwegian Standard Classification of Education [[26], p. 7–8]. Intermediate education includes code 3, 4 and 5. Lower education includes compulsory education, codes 1 and 2.

Characteristics of net sample. Data are presented as percent. (N).

The bivariate logistic regression analysis (table 5, table 6, table 7) indicate that for all three measures, children from families living in the region with the highest uptake (Akershus) were significantly healthier than were children in other counties, although the differences were small. Children from families living in regions with a lower level of uptake did not differ from those in the reference group. Overall, health conditions were better for children from families with a higher level of parental education. The results in Tables 5 and table 6 also show a tendency for members of the association to have children with a more severe condition. The results could not verify that families attending a private specialist had better health conditions.

**Table 5:** Probability of reporting a positive self assessed health condition^1^.

	A Bivariate analysis		B Multivariate analysis	
	Odds ratio	95% confidence interval	Adjusted odds ratio	95% confidence interval
*Level of uptake in county*				
Hi level (Akershus)	1.44*	1.06–2.40	1.13	0.79–2.17
Medium/hi	Reference	Reference	Reference	Reference
Medium/low	1.22	0.81–1.86	1.26	0.81–1.97
Low	1.12	0.70–1.79	1.22	0.76–1.96
				
*Centrality*				
Least central municipalities	0.91	0.56–1.48	1.08	0.64–1.84
				
*Educational background *^2^				
Both parents higher education	3.18**	1.91–5.23	3.15**	1.86–5.36
One of parents higher education	1.70*	1.14–2.52	1.75**	1.17–2.63
Parents with intermediate education	Reference	Reference	Reference	Reference
				
Both parents lower education	0.99	0.62–1.52	0.90	0.57–1.43
				
*Regular source of care*				
Using private specialist care	1.30	0.94–1.76	1.13	0.76–1.68
				
*Social network and social participation*				
Membership in the Norwegian Association for Asthma and Allergy	0.66**	0.48–0.88	0.62**	0.44–0.87
Participation in activities related to asthma arranged by patient organizations	0.89	0.65–1.23	1.03	0.73–1.45
Families with a close friend or relative who is a doctor	1.84*	1.01–3.35	1.20	0.62–2.33

Logistic regression analysis. Odds Ratio. 95% confidence interval

* p < .05, p < .01 **

^1 ^Self reported health, Very good = 1, Good/Fair/bad/Very bad = 0

^3^Higher education includes first stage of tertiary education and postgraduate education, code 7 and 8 in The Norwegian Standard Classification of Education [[28], p. 7–8]. Intermediate education includes code 3, 4 & 5. Lower education includes Compulsory education, code 1 & 2.

**Table 6:** Probability of reporting less than 4 episodes of heavy breathing or wheezing in chest during previous 12 months^1^.

	A Bivariate analysis		B Multivariate analysis	
	
	Odds ratio	95% confidence interval	Adjusted odds ratio	95% confidence interval
*Level of uptake in county*				
Hi level (Akershus)	1.54**	1.16–2.05	1.44*	1.01–2.05
Medium/hi	Reference	Reference	Reference	Reference
Medium/low	0.91	0.68–1.22	0.95	0.69–1.31
Low	0.79	0.57–1.11	0.73	0.51–1.04
				
*Centrality*				
Least central municipalities	1.19	0.85–1.65	1.59*	1.11–2.29
				
*Educational background *^3^				
Both parents higher education	1.45	0.89–2.15	1.45	0.90–2.31
One of parents higher education	1.15	0.81–1.48	1.14	0.83–1.56
Parents with intermediate education	Reference	Reference	Reference	Reference
				
Both parents lower education	0.66**	0.50–0.95	1.44*	1.01–2.50
				
*Regular source of care*				
Using private specialist care	1.27*	1.01–1.60	1.08	0.81–1.44
				
*Social network and social participation*				
Membership in the Norwegian Association for Asthma and Allergy	0.67**	0.54–0.83	0.64**	0.50–0.82
Participation in activities related to asthma arranged by patient organizations	0.74**	0.59–0.93	0.82	0.63–1.05
Families with a close friend or relative who is a doctor	1.05	0.62–1.76	0.83	0.46–1.48

Logistic regression analysis. Odds Ratio. 95% confidence interval

* p < .05, p < .01 **

^1^Less than 4 episodes = 1, 4 or more episodes = 0.

^2^Higher education includes first stage of tertiary education and postgraduate education, code 7 and 8 in The Norwegian Standard Classification of Education [[26], p. 7–8]. Intermediate education includes code 3, 4 & 5. Lower education includes Compulsory education, code 1 & 2.

**Table 7:** Probability of reporting sleeping disorders^1^.

	A Bivariate analysis		B Multivariate analysis	
	
	Odds ratio	95% confidence interval	Adjusted odds ratio	95% confidence interval
*Level of uptake in county*				
Hi level (Akershus)	0.72*	0.55–0.96	0.77	0.55–1.08
Medium/hi	Reference	Reference	Reference	Reference
Medium/low	1.13	0.88–1.46	1.10	0.83–1.45
Low	0.80	0.59–1.06	0.80	0.59–1.08
				
*Centrality*				
Least central municipalities	0.75	0.54–1.04	0.71	0.51–1.01
				
*Educational background *^2^				
Both parents higher education	0.47**	0.30–0.81	0.50**	0.30–0.84
One of parents higher education	0.73*	0.57–0.99	0.77	0.57–1.04
Parents with intermediate education	Reference	Reference	Reference	Reference
				
Both parents lower education	1.66**	1.16–1.96	1.55**	1.19–2.03
				
*Regular source of care*				
Using private specialist care	0.90	0.73–1.11	0.94	0.73–1.23
				
*Social network and social participation*				
Membership in the Norwegian Association for Asthma and Allergy	1.13	0.92–1.37	1.19	0.95–1.48
Participation in activities related to asthma arranged by patient organizations	1.16	0.95–1.42	1.12	0.89–1.39
Families with a close friend or relative who is a doctor	0.73	0.44–1.19	1.02	0.59–1.76

Logistic regression analysis. Odds Ratio. 95% confidence interval

* p < .05, p < .01 **

^1^1 or more nights pr week of interrupted sleep because of cough during the last 12 months = 1, less than I or more times a week = 0.

^2^Higher education includes first stage of tertiary education and postgraduate education, code 7 and 8 in The Norwegian Standard Classification of Education [[26], p. 7–8] Intermediate education includes code 3, 4 & 5. Lower education includes Compulsory education, code 1 & 2.

A comparison of the estimates in columns A and B indicate that a part of the Akershus effect can be accounted for by other variables related to the families. For outcomes such as self-assessed health condition and the probability of reporting sleeping disorders, the statistical effect of living in Akershus was no longer significant when centrality, regular source of care, parental education, social networks and social participation were controlled for. With regard to the probability of reporting episodes of heavy breathing, the Akershus effect was less significant in the multivariate analysis, and the odds ratio decreased slightly from 1.54 to 1.44.

Table 8 presents the results of logistic regression exploring the association between family variables and experience in delay in applying for benefit. Families from Akershus were significantly less likely to have experienced an excessively long processing of the application (column A). Families living in regions with a lower level of uptake did not appear to be more likely to experience delays. As noted previously in Table 3, families in Akershus were more often informed by a doctor or staff at a hospital about their rights to benefits. Families who received information from these sources were less likely to have experienced delays in the processing of the application (column A). This finding indicates that a contextual effect is in operation. Families using a private practice specialist were less likely to report delay. However, the significance of this finding was relatively weak compared with the other associations in column A. No experiences with delay with the application process were also clearly associated with having a close friend or family member who was a doctor. A clear pattern was also seen for educational background. Families with parents with a higher education level reported were less likely to report delays in the application process.

**Table 8:** Probability of not reporting a delay in the processing of the application^1 ^Logistic regression analysis.

	A Bivariate analysis		B Multivariate analysis	
	
	Odds ratio	95% confidence interval	Adjusted odds ratio	95% confidence interval
*Level of uptake in county*				
Hi level (Akershus)	2.12**	1.60–2.78	2.05**	1.44–2.93
Medium/hi	Reference	Reference	Reference	Reference
Medium/low	0.86	0.64–1.16	0.96	0.69–1.33
Low	1.29	0.86–1.60	1.22	0.87–1.70
				
*Centrality*				
Least central municipalities	0.85	0.60–1.19	1.05	0.73–1.54
				
*Regular source of care*				
Using private specialist care	1.26*	1.01–1.57	0.88	0.66–1.17
				
*Source of information about the possibility of applying for benefits *(family and other informal sources = reference category)				
Informed by a physician	3.00**	2.00–3.14	2.52**	1.96–3.25
Informed by a nurse or social worker	2.94**	1.48–2.80	2.52**	1.77–3.58
				
*Educational background*^2 ^(parents with intermediate education = reference category)				
Both parents higher education	2.06**	1.41–3.26	1.61*	1.02–2.55
One parent higher education	1.56**	1.19–2.10	1.60**	1.18–2.12
Parents with intermediate education	Reference	Reference	Reference	Reference
Both parents lower education	0.67	0.57–1.06	0.84	0.61–1.16
				
*Social network and social participation*				
Families with a close friend or relative who is a doctor	2.24**	1.42–3.53	1.42	0.84–2.43

Odds Ratio. 95% confidence interval

* p < .05, p < .01 **

^1^Families that responded "no" to the expression "Are you of the opinion that the application for benefit should have been sent before?"

^2^Higher education includes first stage of tertiary education and postgraduate education, code 7 and 8 in The Norwegian Standard Classification of Education [[26], p. 7–8]. Intermediate education includes code 3, 4 & 5. Lower education includes Compulsory education, code 1 & 2.

Column B of Table 8 presents the multivariate model that included all the variables. Using private specialist care or having a close friend or relative who is a doctor was no longer significant in this analysis. In the multivariate model, the significance and odds ratios for families with a higher education level decreased. The most significant characteristic of families that did not report a delay in the application process was that they were informed about their rights by a physician, nurse or social worker at a hospital. The odds ratio of reporting no delays by families in Akershus decreased from 2.14 (column A) to 2.05 (column B) compared with families in the reference group living in regions with a medium or high uptake of benefits. The implication is that the lower likelihood of delays in processing the application for respondents from Akershus can be accounted for only partly by the other variables included in the empirical model. Column A of Table 8 shows that a compositional element was also involved; that is, families with parents with a higher education level tended to report less frequent delays in the processing of the application.

## Discussion

The families with a higher level of parental education receiving BA-benefits reported a consistently better health condition of their children. These results indicate the existence of a possible "Matthew effect" in the relationship between parental education level and delays in the processing of the application for benefits. A systematically lower threshold for receiving benefits seems to exist for families with higher parental education level. To some extent, a high uptake in the county of Akershus can be explained by the compositional effect because families with highly educated parents in the sample have healthier children and are less likely to report delays in the application process. The empirical design of this study makes it difficult to distinguish the effect of education from other variables and to assess the direction of causality involved. It is possible that highly educated parents are better informed about their options, but it is also possible that health personnel are more attentive to the needs of parents with higher education levels.

Lay referral systems play an important role as a source of information about benefits. However, this study could not confirm that members in patient organizations had more positive experiences with the application process. Members of patient organizations tended to have children with more severe conditions, which might suggest that families with children with more severe asthma are more likely to seek membership.

Being personally acquainted with a physician had a positive influence on how parents experienced the application process. Although not significant in the multivariate model, this may reflect a contextual effect in Akershus.

Private practice paediatricians were identified as an institutional context of particular relevance for asthmatic children and their families. The use of private specialist care was relatively common in Akershus, irrespective of parental educational background; a finding also reported in a British context [29]. Information from official registers of specialist health care personnel confirmed an uneven geographic distribution of this particular source of care. Ten of 19 counties did not have any private practice paediatricians, whereas Akershus had the highest coverage of paediatricians during the investigation [23]. Despite the dominance of this particular context, the statistical effect was relatively modest. An explanation based on a private institutional context is only one of several contributing factors. The crucial factor identified in this study is that physicians and health and social care workers within hospitals tend to inform families about their social rights. Irrespective of the social background of the parents, after controlling for educational background in the multivariate analysis, families in Akershus were given more information about their rights by the health services than were other families.

Although the analysis could explain some of the high level of uptake in the county of Akershus, the evidence does not further our understanding of why some counties had a very low level of benefits.

The applied empirical design has several shortcomings. A common problem in these types of investigations is the lack of relevant measures of need [3]. Additional analysis from the National Health Survey could not confirm a higher morbidity from asthma in Akershus. The survey conducted in 1995 included a representative net sample of 2,316 children aged younger than 17 years [30]. The child and adult representative were asked whether they had any present or previous experience with asthma or allergy. An analysis of this material (data not shown) showed that 34% of the children in Akershus had asthma or allergy or had previous experience with these conditions. The national average was 30%.

For the present study, a measure of the number of people eligiblefor benefits would have been useful. However, if such measures had existed, benefits could simply have been granted directly without a complex procedure involving medical documentation and final approval by the local insurance administration. Ideally, the research design should have included two additional groups: families with children with a chronic asthma condition who do not apply for benefits, and families that have applied for benefits but were unsuccessful in their attempt. Information about the extent of these two groups is not known. It seems plausible that the same types of mechanisms that were present in the analysis of parents' perceived delay in receiving benefits could also be present in families whose applications were declined or among families that did not attempt to apply for benefits. If so, the inequalities are underestimated.

Few differences between parents with low education and intermediate education were observed. The reliability of this finding may also be questioned. From the relevant available information about non-responders, families with low parental education level were less likely to respond (response rate of 61%). Although a reasonable response rate, this finding exemplifies a general limitation of the survey methodology – a relative under-representation of vulnerable groups. The question is whether non-responders with a low education level would have responded to the questionnaire in the same way as the responders did. My suggestion is that non-responders with low education levels are less likely to apply for benefits and that variations in the response rate can underestimate differences between responders with different education levels. In addition, the study was based on a sample in which everyone was granted benefits. It seems likely that some parents, even those with severe asthmatic child, may have been unaware of their rights; little is known about the size and characteristics of such a group. However, an empirical design based on a representative sample would be costly to establish and subjected to the same problems with regard to non-response bias.

Another limitation follows from the fact that the survey was conducted in 1999. The regional distribution of relevant professional groups may have changed over time, and well as their overall knowledge about cash benefits. Internet access have also changed dramatically in the period since the data was collected, making information about cash benefits more accessible. The extent to which these changes have affected the social and regional patterns in uptake is difficult to assess, and requires further research.

Systematic studies of children with chronic disease and their use of health-related benefits are hard to find. Pinch observed that researchers of regional inequalities "have tended to look at spatial variations in the direct provision of welfare services such as schools, hospitals, day-care centres and social services rather than the income maintenance schemes of the welfare state, since the latter tend to be invariant throughout nation" [[31], p. 21]. A similar position is evident in Elster's [32] effort to develop a theoretical and conceptual framework to describe and explain how institutions allocate goods and burdens. In Elster's view, most of the attention is directed to what is termed "local re-distributive policies". These are processes that include the allocation of scarce resources, such as highly specialized medical treatments or access to higher education, and that involve actors with considerable personal autonomy to design and implement their preferred scheme. In contrast, "global re-distributive policies" are designed at the level of national governments, are intended to compensate for various forms of illness and typically take the form of cash transfers. The assumption is that the scope for local variation is limited in the latter type of allocation processes.

The findings reported above contradict the opinion that the distributional outcomes of cash benefit schemes more or less reflect the objectives of the policy makers. According to Lipsky [14], a general stance toward the information provided by official registers seems to be more appropriate. Lipsky claims that "Client statistics may not indicate much about the objective needs of the client population but they reflect a great deal about the organizations that formally cater to those needs" [[14], p. 92]. This perspective is similar to that taken by two relevant British studies. A recent investigation of the uptake of health-related cash benefits concluded that geographic variation in the uptake of benefits is influenced by factors unrelated to morbidity and that uptake is lower in areas with higher proportions of ethnic minority populations [6]. Hansell and collaborators [33] compared the geographic patterns of asthma using several sources of data and concluded that these patterns were not consistent across data sources and that healthcare use might not reflect underlying geographic variations in the severity and prevalence of disease.

## Conclusion

Scandinavian welfare states have always given high priority to redistribution and equality. Major contributions to research in the field of comparative welfare states note that welfare states should be judged by what they actually do rather than by the amount of resources they allocate to welfare [34,35]. It has been proposed that the performance of health services should be measured by mortality from specified diseases for which death is avoidable given appropriate medical intervention. Using measures of "avoidable mortality" when assessing differences *between *nations shows that social democratic welfare states are high performers, as expected [36]. However, focusing on socio-economic variations in mortality *within *nations shows that inequalities in Scandinavian welfare states are, surprisingly, ranked among the highest in Western Europe [37]. Although health service performance cannot necessarily be inferred directly from aggregate studies of avoidable mortality [38], this paradox indicates some discrepancy between the reality and the reputation of Scandinavian welfare states. The findings reported by Mackenbach [37] are also compatible with the findings of the present study. One can argue that, in an institutional model based on universal programmes, results like the ones of this study are a likely outcome. People with resources and social networks are more likely to obtain the most out of services that are available to all. In the present case, it seems plausible to expect that granting cash benefits only to families with incomes below a certain income limit would result in a more equal geographic and social distribution. The common argument against such a strategy is that excluding the middle classes from important welfare measures may limit political support for further development of generous welfare measures [39]. However, this does not mean that a better fit between the activities of the street-level bureaucracy of universal welfare states and the population they are supposed to serve is unattainable.

A white paper on strategies aimed at reducing social inequalities in health was recently issued by the social democratic coalition [40]. In the white paper, priority is given to ensuring that all children have equal opportunities regardless of their parents' education and geographical identity [[40], p. 9]. High-quality services for children across the social divide are stated as important means of achieving equity ambitions. The white paper also recognizes that research on both children and geographic perspectives are lacking in the Norwegian context. This is explained by the presumable shortage of available core indicators because children do not have an income and have not yet completed their education, and that commonly used indicators of health such as self-assessed health and mortality are less suited to addressing inequalities among children [[40], p. 90]. However, to refine the implementation of a welfare policy, a better understanding of the processes that generate the outcomes of the various welfare schemes and services is required. This can be achieved by adopting a "micro" approach to the study of outcomes of welfare policy that integrates both the social and geographic perspective. Future studies should address factors relevant to the health care provision at lower regional levels, such as the balance of public to private care and characteristics of the welfare professionals who process claims. Approaches that identify regional and social characteristics of groups that are likely to have applications for benefits turned down, or are likely to be eligible but not apply, could represent a fruitful future research agenda.

## Competing interests

The author declares that they have no competing interests.
